# 893. The Anatomy of a Burn Center: From Survival Units to Multidisciplinary Survivorship Hubs

**DOI:** 10.1093/jbcr/irag033.379

**Published:** 2026-04-06

**Authors:** Eva Murphy, Jonathan E Schoen, Jeffrey E Carter, M Victoria P Miles

**Affiliations:** Burn Center at University Medical Center, New Orleans, LA; UCI Health Regional Burn Center, New Orleans, LA; University Medical Center Burn Center, Louisiana State University Health Sciences Center, New Orleans, LA; University Medical Center Burn Center, Louisiana State University Health Sciences Center, Department of Surgery, New Orleans, LA

## Abstract

**Introduction:**

Since the first civilian burn unit was established in 1947, burn centers in the United States (US) have transformed from small survival-focused wards into verified, multidisciplinary survivorship hubs. While early growth was marked by the proliferation of centers, the defining feature of modern burn care is the integration of diverse specialties and formal verification standards.

**Methods:**

We conducted a narrative historical review (1947–2025) using PubMed searches, American Burn Association (ABA) directories, ABA/American College of Surgeons (ACS) verification criteria, contributor data, and institutional histories. Studies and reports quantifying the number of burn centers, bed capacity, verification status, or team composition were included. Data were extracted and synthesized across five eras: founding (1947–1969), expansion (1970s–1980s), consolidation (1990s–2000s), verification (2010s), and contemporary (2020s).

**Results:**

In 1947, the Evans–Haynes Burn Center marked the nation’s first civilian unit; by 1969, 32 US burn centers were identified. Rapid expansion in the 1970s-1980s produced >100 centers, with approximately 40% of burns treated in specialty facilities. By 1993, 137 centers were active, consolidating to 125 by 2007 as average beds per unit rose from 11 to 14. The 2010s introduced ABA/ACS verification criteria, mandating surgical coverage, rehabilitation, nutrition, and psychosocial services. In 2019, 133 centers existed, 66 verified; by 2023, 113 contributed to BCQP. Modern burn centers now function as multidisciplinary ecosystems, integrating surgery, critical care, nursing, rehabilitation, nutrition, and psychology.

**Conclusions:**

The number of U.S. burn centers plateaued in the 2000s, but their complexity deepened with verification and multidisciplinary care, reshaping outcomes beyond survival to encompass function, mental health, and reintegration. Mass-casualty events have served as inflection points that exposed weaknesses and forced innovation: Coconut Grove (1942) revealed the lethal toll of airway injury, the Beverly Hills Supper Club fire (1977) underscored limits of regional capacity, and the Station Nightclub Fire (2003) tested modern transfer protocols. Most recently, the 2025 Bourbon Street attack highlighted the need for coordinated multidisciplinary disaster response. From Coconut Grove to Bourbon Street, each disaster transformed burn care into a system that no longer stops at survival but builds a pathway toward recovery and reintegration - not just to save lives, but to return them.

**Applicability of Research to Practice:**

Understanding this historical trajectory of US burn care highlights how multidisciplinary collaboration has become the cornerstone of burn survivorship. These lessons highlight the need to continue expanding verification, strengthening access, and investing in survivorship programs, ensuring burn centers are equipped to meet the full spectrum of recovery.

**Funding for the study:**

N/A.

